# Identification and Management of Thyroid Dysfunction Using At-Home Sample Collection and Telehealth Services: Retrospective Analysis of Real-World Data

**DOI:** 10.2196/43707

**Published:** 2023-05-30

**Authors:** Kathleen M Gavin, Daniel Kreitzberg, Yvette Gaudreau, Marisa Cruz, Timothy A Bauer

**Affiliations:** 1 Everly Health, Inc Austin, TX United States; 2 Division of Geriatric Medicine Medicine Department of Medicine University of Colorado Anschutz Medical Campus Aurora, CO United States; 3 Division of General Internal Medicine Department of Medicine University of Colorado Anschutz Medical Campus Aurora, CO United States

**Keywords:** telemedicine, consumer-initiated testing, dried blood spot, adult, human, access to care, monitoring, telehealth, home-based, at home, laboratory test, blood test, thyroid, screening

## Abstract

**Background:**

Programs aimed at modernizing thyroid care by pairing at-home sample collection methods with telehealth options may serve an important and emerging role in thyroid care.

**Objective:**

The primary objective of this analysis was to evaluate telehealth use, demographics, and clinical characteristics of a cohort of consumer-initiated at-home laboratory thyroid test users who were also offered the option of follow-up telehealth consultations.

**Methods:**

This was a retrospective analysis of real-world data from a deidentified consumer database of home-collected, mail-in thyroid tests used from March to May 2021 (N=8152). The mean age was 38.6 (range 18-85) years, and 86.6% (n=7061) of individuals identified as female.

**Results:**

In total, 7% (n=587) of test takers fell into a thyroid dysfunction category (overt hypothyroidism: n=75, 0.9%; subclinical hypothyroidism: n=236, 2.9%; overt hyperthyroidism: n=5, 0.1%; and subclinical hyperthyroidism: n=271, 3.3%). Overall, 12% (n=984) of the overall sample opted into a telehealth consultation, with 91.8% (n=903) receiving a nontreatment telehealth consultation and 8.2% (n=81) receiving a treatment telemedicine consultation. Furthermore, 16% (n=96) of individuals with overt or subclinical thyroid dysfunction engaged in telehealth consultations. The majority of treatment consultations (59.3%, n=48) were conducted with people reporting a history of thyroid issues, with 55.6% (n=45) of people indicating wanting to discuss their current thyroid medication and 48% (n=39) receiving a prescription medication.

**Conclusions:**

The combination of at-home sample collection and telehealth is an innovative model for screening thyroid disorders, monitoring thyroid function, and increasing access to care, which can be implemented at a large scale and across a wide range of age groups.

## Introduction

Telehealth is an attractive option for modern health care as it has the potential to improve many barriers to care including accessibility, quality, and cost [1]. Research suggests patients with a wide variety of health problems, including thyroid disorders, are generally satisfied with health management via telemedicine [2-4]. Although to date its use has been limited in scope, telemedicine has the potential to serve an important and emerging role in managing some thyroid disorders [5,6], particularly in combination with laboratory testing. Detection and diagnosis of thyroid disorders are important public health issues. According to the American Thyroid Association, nearly 20 million Americans currently have some form of thyroid disease, and over 12% of individuals in the United States will develop a thyroid condition within their lifetime [7].

It is common for patients to request specific tests from their physicians [8-10]. More recently, commercial avenues have become available for consumers to pursue testing. In consumer-initiated testing (CIT), individuals order a laboratory test based on their health-related needs and goals, which is then reviewed and authorized by a physician. In some cases, the associated sample collection can be completed by the consumer at home. Combining at-home sample collection with certified laboratory-based testing and telehealth services can provide an accessible and convenient platform for consumers to initiate management of their health. In resource-limited settings or during periods in which travel is restricted or health care resources overwhelmed, these approaches may be particularly appropriate [11,12].

Studies suggest that those with symptoms of thyroid dysfunction may have lower quality of life and higher rates of clinical comorbidities such as cardiovascular diseases and diabetes [13,14]. Individuals with hypothyroidism commonly experience fatigue, depression, impaired memory, and irregular menstrual periods [15,16], while individuals with hyperthyroidism may experience unintentional weight loss, rapid or irregular heartbeat, anxiety, nervousness, fatigue, and sleep problems [17]. Symptoms of hypo- or hyperthyroidism may lead an individual to pursue CIT for thyroid health evaluation. However, limited data are available regarding novel programs aimed at modernizing thyroid care using a combination of CIT with at-home collection methods and telehealth offerings. The primary objective of this retrospective analysis was to evaluate telehealth use, demographics, and clinical characteristics of a cohort in a pilot program pairing consumer-initiated home-collection and laboratory-based testing with the option for follow-up telehealth consultations.

## Methods

### Ethical Considerations

This is a retrospective data analysis of real-world data. The project was deemed exempt from review by the WCG institutional review board because it does not meet the definition of human subjects research (federal regulation 45 CFR 46.102).

### Data Collection and Testing

This analysis included deidentified data from a consumer database of at-home collected, mail-in thyroid tests (Everlywell, Inc). Consumers had independently purchased thyroid test collection kits. The cost of the test kit was either paid out of pocket by the consumer or was a qualifying health savings account or flexible savings account expense, if applicable. Data from consumers aged 18 years and older and with valid test results collected between March 1 and May 12, 2021, were eligible for inclusion in the analysis. Age, sex, and zip code were reported at purchase. Truncated zip codes were used to categorize US census regions.

The thyroid test collection kits included all materials necessary and instructions for self-collection of dry blood spot (DBS) samples, including a link to an instructional video on how to properly perform the finger stick, the self-collection procedure, and how to evaluate the sample sufficiency. Following collection, DBS samples were shipped, using prepaid shipping materials, to Clinical Laboratory Improvement Amendments (CLIA)– or College of American Pathologists (CAP)–certified Everlywell Diagnostics or CLIA-certified ZRT Laboratory for analysis of thyroid-stimulating hormone (TSH), free thyroxine (T_4_), free triiodothyronine, and thyroid peroxidase antibodies. Only TSH and free T_4_ were included in this analysis. The laboratory-defined reference range for TSH was 0.55 to 4.78 mIU/L at Everlywell Diagnostics and 0.5 to 3.0 mIU/L at ZRT. The reference range for free T_4_ was 0.7 to 2.5 ng/dL at both laboratories. Kits processed at ZRT with TSH results below 0.2 mIU/L were reported as “<0.2” (n=14) and included in the analysis as 0.1 mIU/L. Test results otherwise outside of the limits of detection and tests that did not result were excluded from the analysis.

Notification emails were sent to patients when their results were available. A digital platform displayed biomarker results in the context of the laboratory-defined reference ranges and included an opt-in link for a telehealth follow-up with a health care professional for interested individuals. The telehealth intake form included an eligibility questionnaire. If eligibility requirements for a treatment consultation were not met, patients were given the option to continue with a nontreatment telehealth consultation. A nontreatment telehealth consultation involved the opportunity to discuss test results with a health care professional, but prescriptions were not available. Individuals were referred to in-person care if appropriate.

The telemedicine treatment consultations were focused on hypothyroidism, and eligibility included having a TSH value ≥4 mIU/L or currently taking any thyroid medication. Exclusion criteria included pregnant, planning to become pregnant, or within 1 year post partum; Turner syndrome; Down syndrome; multiple sclerosis; ischemic heart disease; goiter or suspected goiter; disorders affecting pituitary gland or hypothalamus; endocrine dysfunction as a result of traumatic brain injury; ongoing cancer treatment; HIV; received contrast in the past 6 weeks; or currently taking lithium carbonate, amiodarone, aminoglutethimide, interferon ɑ, thalidomide, betaroxine, or stavudine. Telemedicine treatment consultations involved speaking with a physician about the test results, with the opportunity to obtain a prescription if medically appropriate.

Patients with a TSH level of >10 mIU/L were contacted via telephone by a patient care department representative. During the phone call, patients were notified that they received an abnormal TSH result and that a physician was available to answer questions and help manage their thyroid health.

In total, 23 different physicians provided both treatment and nontreatment consultations including a median of 84 consultations per physician, ranging from 1 to 227 consultations per physician. Physician specialties included 52.2% (n=12) family medicine, 30.4% (n=7) internal medicine, and 13.0% (n=3) emergency medicine and/or preventative medicine.

### Statistical Methods

Descriptive statistics were used to summarize the study sample including demographics, prevalence of individuals within thyroid function categories, range of biomarker values within thyroid function categories, the proportions of individuals who opted for treatment or nontreatment consultations, and prescription status. Descriptive statistics include numbers and percentages for categorical variables and medians and IQRs or means and SDs for continuous variables. All statistics were completed in RStudio (version 4.0.3; RStudio, PBC) for macOS Catalina.

## Results

The analysis included test results from 8152 individuals (Table 1). Over half of the sample was 30 to 44 years, with nearly 90% (n=7061) identifying as female. In total, 7% (n=587) of test takers fell into a thyroid dysfunction category, and the remaining 93% (n=7565) of the sample was categorized as euthyroid.

The TSH and free T_4_ values among thyroid function categories are in Table 2. As expected, the range of TSH and free T_4_ values correspond with criteria for identifying each thyroid function category. Individuals in the euthyroid category had a median TSH level of 1.45 (IQR 0.9) mIU/L and mean free T_4_ value of 12.5 (SD 3.7) pmol/L. In general, the difference in proportion between males and females within each thyroid function category tracked with overall test usage, with females making up ≥83% of those in each category.

Hypothyroid categorization aligned with increased prevalence in older age, with those ≥45 years of age making up 52% (n=39) of individuals categorized as overt hypothyroid and 36% (n=86) of those categorized as subclinical hypothyroid (Table 2). However, as would be expected, 80% (n=4) of individuals in the overt hyperthyroidism category and 62% (n=168) in the subclinical hyperthyroid category were <45 years of age.

In total, 12% (n=984) of the overall sample opted into any telehealth consultation (range by thyroid function categorization 10.6%-21.3%; Table 2). Among patients who received a TSH result ≥4 mIU/L (n=376), 13.8% (n=52) opted for a telehealth consultation and 88.5% (n=46) of those were treatment consultations. In addition, of those with overt or subclinical thyroid dysfunction (n=587), 16.4% (n=96) engaged in telehealth consultations, 6.8% (n=40) of those were treatment consultations. The nontreatment telehealth consultation was primarily used by individuals in the subclinical and overt hyperthyroid (Table 2) and euthyroid (n=847, 11.2% of category) categories who did not qualify for a treatment consultation.

Table 3 describes the telehealth users by consultation type. In total, 21% (n=205) of those engaging in any telehealth consultation had a pre-existing thyroid condition, while the other 79% (n=779) were de novo. The most widely reported reason for opting into a telehealth consultation overall was to discuss test results (n=875, 88.9%), followed by wanting to understand how to treat thyroid dysfunction (n=526, 53.5%). While individuals with a history of thyroid issues only represented 17.4% (n=157) of nontreatment telehealth consultations, they represented over half (n=48) of the treatment telemedicine group. There was greater representation from older age groups opting into treatment consultations compared to the overall sample, which was reflective of their greater proportion in the hypothyroid categories. Medication was of importance to those in the treatment telemedicine group; 55.6% (n=45) indicated being interested in discussing their current medication and 48.1% (n=39) received a prescription medication, 43.6% (n=17) of whom indicated starting a medication as the reason for wanting a consultation. Notably, half of those who received a prescription medication reported a history of thyroid issues (n=21). Of the 83 individuals who were currently taking a thyroid medication and wanted to discuss potential changes, 31.3% (n=26) had biomarker levels indicative of thyroid dysfunction, 54.2% (n=45) received a telemedicine treatment consultation, and 15.6% (n=13) received a prescription medication from that consultation.

The number of reported chronic conditions was similar among nontreatment and treatment telehealth patients (Table 3). The most common chronic condition among all telehealth patients was anemia (n=70, 7.1%), followed by celiac disease (n=18, 1.8%) and type 1 diabetes (n=4, 0.4%). The most common family history condition was hypothyroidism, followed by Hashimoto thyroiditis (ie, autoimmune hypothyroidism) and hyperthyroidism (Table 3).

The average number of symptoms reported among all telehealth users was 6 (SD 3; Table 3). The most common symptom reported was fatigue and weakness (n=768, 78%), which was most often coreported with weight gain (n=621, 63.1%), intolerance to cold (n=444, 45.1%), depression (n=438, 44.5%), and impaired memory (n=437, 44.4%).

**Table 1:** Demographic characteristics (N=8152).

Participant characteristics		Values,^a^ n (%)
**Age (years)**		
	18-29	1661 (20.4)
	30-44	4449 (54.6)
	45-55	1363 (16.7)
	56-69	585 (7.2)
	≥70	94 (1.2)
**Sex**		
	Male	1091 (13.4)
	Female	7061 (86.6)

^a^Number and percentage of all thyroid test users.

**Table 2:** Clinical thyroid categories and telemedicine opt-ins.

		Overt hypothyroidism (N=75, 0.9%)	Subclinical hypothyroidism (N=236, 2.9%)	Overt hyperthyroidism (N=5, 0.1%)	Subclinical hyperthyroidism (N=271, 3.3%)
TSH (mIU/L),^a^ median (IQR)		8.83 (16.91)	5.57 (3.13)	0.32 (0.07)	0.39 (0.21)
Free T_4_ (pmol/L),^b^ mean (SD)		7.3 (0.8)	14.2 (4.2)	35.9 (3.0)	14.4 (4.1)
**Sex, n (%)**					
	Male	7 (9.3)	39 (16.5)	0 (0.0)	27 (10.0)
	Female	68 (90.7)	197 (83.5)	5 (100.0)	244 (90.0)
**Age (years), n (%)**					
	18-29	6 (8.0)	47 (19.9)	0 (0.0)	39 (14.4)
	30-44	30 (40.0)	103 (43.6)	4 (80.0)	129 (47.6)
	45-55	21 (28.0)	50 (21.2)	1 (20.0)	60 (22.1)
	56-69	14 (18.7)	31 (13.1)	0 (0.0)	40 (14.8)
	≥70	4 (5.3)	5 (2.1)	0 (0.0)	3 (1.1)
Nontreatment telehealth consultation, n (%)		1 (1.3)	3 (1.3)	1 (20.0)	51 (18.8)
Treatment telemedicine consultation, n (%)		15 (20.0)	22 (9.3)	0 (0.0)	3 (1.1)
Neither telehealth nor telemedicine consultation, n (%)		59 (78.7)	211 (89.4)	4 (80.0)	217 (80.1)
Received prescription, n (% of treatment telemedicine users)		12 (80.0)	17 (77.3)	0 (0.0)	0 (0.0)

^a^The laboratory-defined reference range for thyroid-stimulating hormone (TSH) was 0.55 to 4.78 mIU/L at Everlywell Diagnostics and 0.5 to 3.0 mIU/L at ZRT.

^b^The reference range for free thyroxine (T_4_) was 0.7 to 2.5 ng/dL at both laboratories.

**Table 3:** Characterization of individuals completing either a nontreatment telehealth or treatment telemedicine consultation.^a^

Demographics			Nontreatment consultation^b^ (N=903, 91.8%)		Treatment consultation^c^ (N=81, 8.2%)		Received Rx^d^ (N=39, 4.0%)		Total (N=984, 100.0%)
Female, n (%)			786 (87.0)		79 (97.5)		38 (97.4)		865 (87.9)
**Age (years), n (%)**									
	18-29	212 (23.5)		11 (13.6)		7 (17.9)		223 (22.7)	
	30-44	510 (56.5)		32 (39.5)		15 (38.5)		542 (55.1)	
	45-55	130 (14.4)		21 (25.9)		10 (25.6)		151 (15.3)	
	56-69	44 (4.9)		14 (17.3)		6 (15.4)		58 (5.9)	
	≥70	7 (0.8)		3 (3.7)		1 (2.6)		10 (1.0)	
**Reasons for consultation, n (%)**									
	Discuss results	834 (92.4)		41 (50.6)		20 (51.3)		875 (88.9)	
	Understand how to treat thyroid dysfunction	497 (55.0)		29 (35.8)		19 (48.7)		526 (53.5)	
	Interested in starting medication	375 (41.5)		20 (24.7)		17 (43.6)		395 (40.1)	
	Currently taking medication and want to discuss if changes are necessary	38 (4.2)		45 (55.6)		13 (33.3)		83 (8.4)	
	Not sure	30 (3.3)		1 (1.2)		0 (0.0)		31 (3.2)	
**Personal history, n (%)**									
	Had personal thyroid history	157 (17.4)		48 (59.3)		21 (53.8)		205 (20.8)	
	Received thyroid surgery, radioactive iodine therapy, or radiation of the head and neck	17 (1.9)		7 (8.6)		3 (7.7)		24 (2.4)	
**Diagnosed with a chronic condition,^e^ n (%)**									
	Any chronic condition	120 (13.3)		10 (12.3)		7 (17.9)		130 (13.2)	
**Family history,^f^ n (%)**									
	Hypothyroidism	367 (40.6)		40 (49.4)		20 (51.3)		407 (41.4)	
	Hyperthyroidism	108 (12.0)		8 (9.9)		4 (10.3)		116 (11.8)	
	Hashimoto (autoimmune) thyroiditis	106 (11.7)		12 (14.8)		6 (15.4)		118 (12.0)	
	Graves disease	41 (4.5)		5 (6.2)		4 (10.3)		46 (4.7)	
Symptoms,^g^ mean (SD)			6.09 (3.39)		5.37 (3.49)		5.00 (2.48)		6.03 (3.41)

^a^Data are the number and percentage of those within a telemedicine category unless otherwise indicated. Individuals could endorse more than one reason for opting into telemedicine consultation.

^b^Nontreatment telehealth consultations were available to all thyroid test takers.

^c^Treatment telemedicine consultation eligibility: thyroid-stimulating hormone value ≥4 mIU/L or currently taking thyroid medications and had no contraindications.

^d^Only patients receiving a treatment telemedicine consultation were eligible to be prescribed thyroid medication.

^e^Chronic conditions included anemia, celiac disease, type I diabetes, and “others.”

^f^The 3 most common family history conditions and Graves disease were included in this table, other conditions included chronic immune thyroiditis and goiter.

^g^Index of the number of symptoms, associated with both hypo- and hyperthyroidism, each patient reported. The most commonly reported symptoms were fatigue and weakness, weight gain, intolerance to cold, depression, impaired memory, and nerve entrapment.

## Discussion

### Principal Findings

This pilot program paired consumer-initiated at-home sample collection and laboratory-based testing with the option for follow-up telehealth consultations. It was used by individuals interested in pursuing new thyroid diagnoses as well as by those with preexisting thyroid disorders that were likely partial to the ability to access care based on individual time and location preferences. In this pilot program, 16% (n=96) of those whose laboratory values were indicative of overt or subclinical thyroid function engaged in telehealth consultations. Of those who opted into a telemedicine treatment consultation (n=81), which was focused on hypothyroidism, nearly 60% (n=48) of them had a personal history of thyroid problems, and over 50% (n=45) of them were taking thyroid medication at the time of testing and wanted to discuss that with a clinician. These numbers highlight patient acceptance and interest in telehealth consultations as an adjunct to consumer-initiated at-home thyroid tests and for ongoing treatment and monitoring of previously diagnosed thyroid disorders.

Studies are lacking on the use of telemedicine as a substitute for in-person visits with a provider for thyroid care. Measurement of TSH is an effective first step in assessment of thyroid function in most patients [18], and although limitations exist, many aspects of the physical examination can be conducted via telehealth with the assistance of the patient such as visualization of the thyroid, eyes, skin, and assessment for tremor [5,12]. Overall, paired with laboratory testing and referral to in-person follow-up procedures for conditions needing specialized attention, telemedicine is becoming a more acceptable option for thyroid care [5,6,19]. This may be particularly true for routine monitoring of thyroid conditions such as hypothyroidism and appointments for prescription refills or dose adjustments where diagnostic testing beyond laboratory values is not necessarily indicated. This aligns with a large proportion of consumers’ reasons for requesting a telemedicine consultation in our sample.

Importantly, it is estimated that 30%-40% of individuals reporting thyroid disease or taking thyroid medications continue to have abnormal TSH levels [20-22]. Indeed, in our sample, 31% (n=26) of those currently on thyroid medications fell into clinical thyroid categories. This emphasizes the importance of continued monitoring in patients taking thyroid medications and for compliance or concerns of misuse. Additionally, repeated laboratory measurements are recommended for thyroid dysfunction diagnosis, particularly of subclinical disease [18,23-27]. However, patients often experience prolonged wait times for appointments to see medical providers [28]. The adult endocrinology subspecialty has a particularly large burden of mismatch between provider supply and patient demand that is forecasted to widen [29,30]. Remote testing models that pair at-home sample collection for certified laboratory testing with telehealth consultations can help to limit the bottleneck for in-person thyroid care providing a convenient, accurate, efficient, and rapid option for diagnosis, routine monitoring, and medication adjustments.

The popularity of CIT offerings is evident with over 8000 customers taking part in this 10-week pilot study with just under 1000 receiving telehealth consultations. We observed on average 16% (n=96) telehealth engagement in individuals falling into 1 of the 4 thyroid dysfunction categories, which may reflect interest yet ongoing hesitancy among patients regarding engagement in telemedicine. In a nationwide survey conducted in 2015, overall, 52% of respondents replied they would be willing to see their own health care provider via telemedicine, while just under 20% were willing to see a different provider from a different organization via telemedicine [31]. This aligns with our program, which involved consultation with a new provider. In addition to preferring to see their own provider, barriers to telemedicine adoption in this model of care include limitations in technology, fear of the use of technology and data security, and wanting the provider to have access to their health records [31,32].

Compared to estimates for the US population [21], this sample was enriched with cases of overt hypothyroidism (0.9% of this sample vs 0.3% of National Health and Nutrition Examination Survey [NHANES]) and subclinical hyperthyroidism (3% vs 0.7%). Individuals choosing to use this program were likely self-selecting given preexisting concerns of thyroid dysfunction. Indeed, those who opted into the telehealth consultations commonly reported a family history of thyroid disorders, had an average of 6 thyroid-related symptoms, and 40% indicated interest in starting a thyroid medication. Thus, while not representative of a truly random population sample, the sample is of adequate size to assess and describe major features among those interested in using CIT to manage thyroid-related health concerns. Overall, there is significant potential for a CIT and telehealth program to provide increased access to care for both early detection and continued monitoring of thyroid dysfunction and facilitate active patient participation in health care decision-making and self-management.

This pilot program surfaced several important considerations for the implementation of future thyroid telehealth programs. First, collecting information regarding the patient’s objectives for testing as well as providing specific instructions for when to perform the test (both time of day as well as in relation to medications or supplements) are important aspects in ensuring the clinical use of the consumer-initiated laboratory test results. Second, referral for repeat laboratory testing for patients obtaining an initial diagnosis or those on thyroid medication with TSH values out of the reference range should be considered [27], especially if the patient has no previous record of TSH measured by DBS. Consistency in the laboratory conducting the measurement should also be controlled, when possible, as results may vary between laboratories. Third, the ability for follow-up visits to be scheduled with the same provider is important for continuity of care and patient comfort and engagement. Finally, the scope of the telemedicine treatment consultations in this pilot program focused on hypothyroidism. Assessment and treatment of hyperthyroidism via CIT and telemedicine programs require further evaluation.

### Limitations

This analysis of CIT data has limitations. Lack of race or ethnicity information and limited representation of males precluded meaningful subanalyses. In addition, thyroid function was based on single, instead of repeat, diagnostic measurement. Multilaboratory testing also poses variance in independently determined reference ranges, which complicates quantitative analyte analysis and thresholds for thyroid function categorization or overt diagnosis were laboratory-specific. Further, there remains contention within the field regarding the appropriate thresholds for normal TSH values, particularly within subgroups such as older adults or specific racial and ethnic groups [33-36]. We used thresholds based on traditional thyroid dysfunction categories and each laboratory’s unique reference ranges. For comparison, the United States Preventive Services Task Force suggests screening and initiation of treatment for TSH values of >10 mIU/L for hypothyroidism and undetectable or <0.1 mIU/L for overt hyperthyroidism [23]. Of note, the average TSH value among patients who received a prescription medication through this program was 9.66 (SD 13.20) mIU/L, just below the United States Preventive Services Task Force recommendations. Unfortunately, it was unknown whether the prescriptions written as part of this program were dose adjustments, refills, or for a new medication. Finally, personal objectives for testing or rate of in-person physician follow-up for those who did not engage in the offered telehealth program were not available. This information, particularly for those falling within clinical categorization for potential treatment, would have been useful in understanding the patient motivations, the barriers to their adoption of telehealth, and in designing new features aimed at improving telemedicine engagement in those needing follow-up care based on their laboratory results.

### Conclusions

These findings demonstrate that expanding options for screening and obtaining ongoing care for monitoring of chronic conditions through consumer-initiated telemedicine programs is an innovative new model that is prime for development. Models that pair at-home laboratory testing with telemedicine consultations within the same provider group for continuity of treatment records are well-positioned to be the leader in this new health care delivery space. We conclude that adjoining CIT, at-home sample collection, and telehealth is a potentially useful and innovative approach for expanding access to early detection of thyroid dysfunction, specifically hypothyroidism, in those experiencing symptoms and monitoring thyroid health in those with an established diagnosis or prescription. Increasing use of posttest telemedicine consultation, particularly for individuals with values indicative of overt hypothyroidism, is a critical next step.

## Abbreviations

CAP: College of American Pathologists
CIT: consumer-initiated testing
CLIA: Clinical Laboratory Improvement Amendments
DBS: dried blood spot
NHANES: National Health and Nutrition Examination Survey
T4: thyroxine
TSH: thyroid-stimulating hormone
